# A referenceless Nyquist ghost correction workflow for echo planar imaging of hyperpolarized [1‐^13^C]pyruvate and [1‐^13^C]lactate

**DOI:** 10.1002/nbm.3866

**Published:** 2017-12-07

**Authors:** Jiazheng Wang, Alan J. Wright, Richard L. Hesketh, De‐en Hu, Kevin M. Brindle

**Affiliations:** ^1^ Cancer Research UK Cambridge Institute University of Cambridge Li Ka Shing Centre Cambridge UK; ^2^ Department of Biochemistry University of Cambridge Cambridge UK

**Keywords:** [1‐^13^C]lactate, [1‐^13^C]pyruvate, EPI, ghosting, hyperpolarized, imaging, metabolism, tumours

## Abstract

Single‐shot echo planar imaging (EPI), which allows an image to be acquired using a single excitation pulse, is used widely for imaging the metabolism of hyperpolarized ^13^C‐labelled metabolites *in vivo* as the technique is rapid and minimizes the depletion of the hyperpolarized signal. However, EPI suffers from Nyquist ghosting, which normally is corrected for by acquiring a reference scan. In a dynamic acquisition of a series of images, this results in the sacrifice of a time point if the reference scan involves a full readout train with no phase encoding. This time penalty is negligible if an integrated navigator echo is used, but at the cost of a lower signal‐to‐noise ratio (SNR) as a result of prolonged T_2_* decay. We describe here a workflow for hyperpolarized ^13^C EPI that requires no reference scan. This involves the selection of a ghost‐containing background from a ^13^C image of a single metabolite at a single time point, the identification of phase correction coefficients that minimize signal in the selected area, and the application of these coefficients to images acquired at all time points and from all metabolites. The workflow was compared in phantom experiments with phase correction using a ^13^C reference scan, and yielded similar results in situations with a regular field of view (FOV), a restricted FOV and where there were multiple signal sources. When compared with alternative phase correction methods, the workflow showed an SNR benefit relative to integrated ^13^C reference echoes (>15%) or better ghost removal relative to a ^1^H reference scan. The residual ghosting in a slightly de‐shimmed B_0_ field was 1.6% using the proposed workflow and 3.8% using a ^1^H reference scan. The workflow was implemented with a series of dynamically acquired hyperpolarized [1‐^13^C]pyruvate and [1‐^13^C]lactate images in vivo, resulting in images with no observable ghosting and which were quantitatively similar to images corrected using a ^13^C reference scan.

**Abbreviations used**
1D/2D/3D: one‐/two‐/three‐dimensional
CSI: chemical shift imaging
DNP: dynamic nuclear polarization
EPI: echo planar imaging
FOV: field of view
FSE: fast spin echo
MRI: magnetic resonance imaging
RF: radiofrequency
ROI: region of interest
SNR: signal‐to‐noise ratio
SpSp: spectral‐spatial
SSIM: structural similarity
TE: echo time

## 1 INTRODUCTION

Dynamic nuclear polarization (DNP) can increase the signal intensities of ^13^C‐labelled molecules by a factor of 10^4^–10^5^, making possible the imaging of tissue metabolism *in vivo*.^1^, ^2^, ^3^, ^4^, ^5^ For example, the imaging of hyperpolarized [1‐^13^C]pyruvate and its downstream metabolic products has been used in cancer research to assess treatment response^6^, ^7^ and disease progression,^8^, ^9^ and in other tissues, such as heart^10^, ^11^ and kidney,^12^ to assess tissue function. The technique has now been translated to the clinic with studies in prostate cancer^13^ and in heart.^14^

A considerable barrier to the imaging of hyperpolarized ^13^C‐labelled metabolites is the short lifetime of the polarization: the [1‐^13^C]pyruvate *T*_1_ *in vivo* is approximately 30 s.^5^ Moreover, each radiofrequency (RF) pulse depletes the polarization, further accelerating signal decay.^5^ Therefore, the determination of kinetic information requires the rapid acquisition of a series of images whilst keeping the number of excitations as small as possible, so as to preserve the hyperpolarized signal. Many pulse sequences have been developed to match these requirements.^15^ These have included echo planar‐based spectroscopic imaging sequences with multiband spatial spectral pulses for the mapping of multiple metabolites,^16^ which was further accelerated using compressed sensing.^17^ Compressed sensing has also been used with chemical shift imaging (CSI) for dynamic studies^18^ and spiral‐CSI has been studied extensively.^19^, ^20^, ^21^ Two‐dimensional (2D) spatial and one‐dimensional (1D) spectral information has been acquired using spatiotemporal encoding after a single excitation pulse,^22^ and we recently proposed a single‐shot three‐dimensional (3D) sequence, which is based on a stack of spiral acquisitions.^23^ Echo planar imaging (EPI) is often used as it is fast (subsecond), minimizes RF pulse exposure (only a single shot is required for a 2D image), produces artefacts that are easier to correct because of the Cartesian k‐space, and there are well‐established methods for image reconstruction. However, a drawback of EPI is Nyquist ghosting in the phase‐encoding direction, caused mainly by eddy currents,^24^ which result in the accumulation of opposite phase errors in odd and even *k*‐space lines.^25^ Fly‐back designs avoid bipolar readout gradients and misalignment between alternate *k*‐space lines^26^; however, they are more prone to geometric distortions and give a lower signal‐to‐noise ratio (SNR).^27^

Nyquist ghosting in hyperpolarized ^13^C EPI can be avoided^26^ or removed by phase correction using a reference scan. In proton magnetic resonance imaging (MRI), this can be achieved by acquiring a reference dataset with the phase‐encoding gradients turned off.^25^ This method, however, is less desirable for hyperpolarized ^13^C MRI because a time point is sacrificed in the acquisition of a full EPI reference scan. Alternatively, a ^1^H reference image can be acquired before the hyperpolarized ^13^C image^27^ or integrated ^13^C reference echoes can be acquired by obtaining extra *k*‐space lines without phase encoding.^28^ We describe here a workflow for Nyquist ghost correction that requires no additional reference acquisition, and compare it with these other methods using both phantom and *in vivo* images.

## 2 METHODS

Half‐field of view (FOV) ghosting in EPI images is induced by: 1) group delays between readout gradients and signal acquisition; and 2) eddy currents in the readout direction.^25^ These result in a zero‐order phase error and a phase error that depends on the position in the readout direction (*x* axis). The overall phase error is:

(1) $$\theta \left(x\right)=\alpha +\mathrm{\beta x}$$

where *α* is the phase error caused by a zero‐order eddy current (e.g. a *B*_0_ shift induced by a gradient pulse) and *β* is the linear phase error caused by the first‐order eddy current in the *x* direction. The phase error accumulated as a result of a group delay is equivalent to the effect of a first‐order short time constant eddy current in the readout gradient and is also included in Equation (1). The phase error θ has alternating polarities in even and odd *k*‐space lines, resulting in Nyquist ghosting^25^:

(2) $$\hat{\rho }=\rho \left(x,y\right)\cos\left[\theta \left(x\right)\right]+\mathrm{i\rho}\left(x,y-\frac{N}{2}\right)\sin\left[\theta \left(x\right)\right]$$

where *ρ* is the ideal image, $\hat{\rho }$ is the reconstructed image, *N* is the number of phase‐encoding steps and *x*/*y* are the coordinates in the ideal image in the frequency‐ and phase‐encoding directions, respectively. In short, the ideal image splits into a real one, at the original location, and an imaginary one, half a FOV away. This model typically holds for images acquired from non‐oblique planes on modern MRI systems, where cross‐term and higher order term eddy currents are limited.

**Figure 1:**
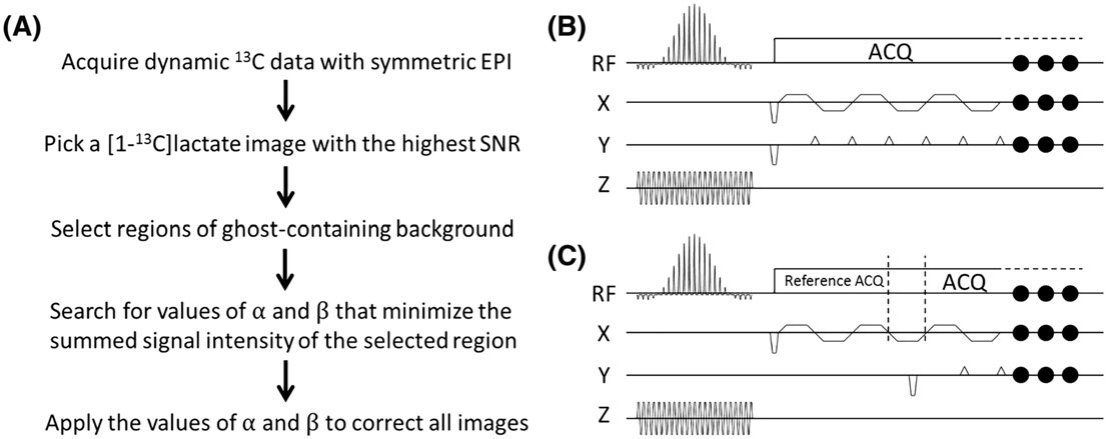
A, Flow diagram outlining the proposed workflow for hyperpolarized ^13^C dynamic magnetic resonance imaging (MRI) with a two‐dimensional Echo planar trajectory. The phase correction coefficients α and β can be searched for in individual images, for example the image with the highest signal‐to‐noise ratio (SNR), or in summed images from the entire time series. The individual slice phase correction coefficients are then applied to the corresponding slices in the other images in the dynamic acquisition. B, Echo planar imaging (EPI) sequence for ^13^C imaging. The ^13^C reference scan used the same sequence, except that the phase‐encoding gradients were turned off. The same gradients for the ^13^C reference scan were also used for the ^1^H reference scan. C, EPI sequence for ^13^C imaging with integrated reference echoes. Three reference echoes, without phase‐encoding gradients, were acquired before the regular acquisition train, with a refocusing lobe in between. RF, radiofrequency

A reference scan can be used to estimate *α* and *β*. Alternatively, an exhaustive search for the zero‐ and first‐order coefficients can be used, which renders a reference scan unnecessary. This concept was proposed initially for proton EPI,^29^ and then expanded^30^ to include a search in the phase‐encoding direction. Our search criterion was to minimize the signal intensity in the ghost‐containing background. This was identified on the basis of prior knowledge of the location of the majority of the ^13^C signal, which was obtained from the proton image. The proposed workflow is illustrated in Figure 1A.

### 2.1 Pulse sequences

The proposed workflow was compared with three other phase correction methods: 1) direct ^13^C reference scan^25^; 2) integrated ^13^C reference echoes^31^; and 3) ^1^H reference scan.^27^ The pulse sequences used in these experiments are shown in Figure 1B, C. The ^13^C imaging sequence acquired a 2D EPI *k*‐space, starting with an excitation pulse and slice selection gradient, followed by a train of 20 bipolar readout gradients and, simultaneously, a train of 19 blipped phase‐encoding gradients. The acquisition matrix was 32 × 32, but only four *k*‐space lines were acquired before the centre of *k*‐space in order to minimize the echo time (TE) and enhance the SNR; hence, only 20 bipolar readout gradients were used (acquiring 62.5% of *k*‐space). For the images acquired *in vivo*, a 15.936‐ms spectral‐spatial (SpSp) pulse (excitation bandwidth, 200 Hz; 1412 Hz between replicate bands) was used for excitation, whereas, for imaging phantoms, a 4‐ms sinc excitation pulse with a bandwidth of 1600 Hz was used. The sinc pulse was designed in Matlab (The Math Works, Natick, MA, USA) using the SLR algorithm^32^ with the rftools toolbox (available online at http://rsl.stanford.edu/research/software.html). The envelope‐pulse and subpulses in the SpSp pulse were also generated using this toolbox, and the envelope‐pulse was then discretely sampled by the subpulse to achieve the desired stopbands between replicated excitation bands. The ^13^C reference scan was acquired with the same imaging parameters, but with the phase‐encoding gradients turned off. For ^13^C imaging with integrated ^13^C reference echoes, three extra readout lobes, with no phase‐encoding gradients, were inserted in front of the regular readout train. Each of the inserted lobes had the same shape as the regular readout lobe. For the ^1^H reference scan, the same excitation pulse (same pulse width and flip angle) and the same readout gradient train (same amplitude and timing) were used as for ^13^C imaging, but with the phase‐encoding gradients turned off. The FOV in the readout direction of the ^1^H reference scan was therefore approximately one‐quarter (*γ*_13C_/*γ*_1H_) of that in the ^13^C image. The slice selection gradient for the ^1^H reference scan was reduced from that used for ^13^C imaging to ensure the selection of the same slice.

### 2.2 Phantom experiments

Phantom experiments were performed on a 7‐T Agilent scanner (Palo Alto, CA, USA) with a ^1^H/^13^C transmit volume coil, ^1^H receive volume coil and a 20‐mm‐diameter ^13^C surface receive coil (RAPID Biomedical GMBH, Rimpar, Germany). The bore size of the gradient coil was 120 mm. The maximum gradient strength was 0.4 T/m and the maximum slew rate was 3000 T/m/s.

A 90° flip angle ^13^C sinc pulse was used in the ^13^C imaging sequence to excite a 10‐mm slice. Proton fast spin echo (FSE) images were acquired with a 256 × 256 matrix from the same slices and same FOVs as used for ^13^C imaging, in order to provide a positional reference. The proposed workflow was compared with the other methods for phase correction under a variety of different acquisition conditions. The receiver bandwidth and echo spacing are given below. The former affects the phase error accumulation and the latter determines image distortion.

#### 2.2.1 Comparison with phase correction using a direct ^13^C reference scan and integrated ^13^C reference echoes

Images were acquired from a cylindrical glass phantom (inner diameter, 7 mm) containing approximately 5 M [1‐^13^C]lactate with a 4 × 4‐cm^2^ FOV, receiver bandwidth of 125 kHz (readout gradient, 0.29 T/m) and echo spacing of 552 μs. To investigate the effect of a restricted FOV, these experiments were repeated with a glass sphere phantom (inner diameter, 17.2 mm) containing approximately 2 M [1‐^13^C]lactate and a 2.4 × 2.4‐cm^2^ FOV. The receiver bandwidth in this case was reduced to 100 kHz (readout gradient, 0.39 T/m), and the echo spacing was therefore extended to 712 μs. To investigate the effect of multiple signal sources, a dual phantom, containing the 7‐mm‐ and 17.2‐mm‐diameter phantoms, was imaged using a 4 × 4‐cm^2^ FOV.

#### 2.2.2 Comparison with ^1^H reference scan under de‐shimmed conditions

The dual phantom described above was used. Images, with a 4 × 4‐cm^2^ FOV, were acquired in a relatively well‐shimmed magnetic field (^13^C linewidth, 21 Hz) and following the imposition of a background *xy* gradient (approximately 0.006 T/m^2^, where the ^13^C linewidth increased to 25 Hz).

#### 2.2.3 Performance of the proposed workflow with different FOVs

To further investigate the tolerance of the proposed workflow to restricted FOVs, images were acquired with different FOVs (20.5, 16.5, 12.5 and 11.5 mm) from a 10.5‐mm‐diameter cylindrical glass phantom filled with 5 M ^13^C‐urea, and the processed images were compared with those phase corrected using the direct ^13^C reference scan method. The receiver bandwidth was 45 kHz, resulting in echo spacings of 931.2, 979.2, 1059.2 and 1091.2 μs, respectively (readout gradient strengths of 0.21, 0.25, 0.34 and 0.37 T/m). All images were acquired using eight averages to ensure that there was a sufficient SNR, even for small FOVs.

### 2.3 *In vivo* imaging

Subcutaneous EL4 lymphomas in C57BL/6J mice^23^ were imaged using the same hardware setup as for the phantoms. Female C57BL/6J mice, which were controls in a larger study, were injected subcutaneously in the lower flank with 5 × 10^5^ EL4 lymphoma cells and the tumours were allowed to grow for 8–11 days, when they were 1–1.7 cm in diameter. The tumours were then placed in the centre of the rigid 20‐mm‐diameter surface coil for the imaging experiments. The mice were fasted for 6–8 h before imaging, as this has been shown to lead to more reproducible lactate labelling.^33^ [1‐^13^C]Pyruvate was hyperpolarized as described previously,^23^ and the imaging pulse sequence was started at the time of injection of 400 μL of 82mM [1‐^13^C]pyruvate. Animal experiments were performed in compliance with a project licence issued under the Animals (Scientific Procedures) Act of 1986. Protocols were approved by the Cancer Research UK, Cambridge Institute Animal Welfare and Ethical Review Body.

The ^13^C images covered a 4 × 4‐cm^2^ FOV, with a receiver bandwidth of 125 kHz, and were from a 6‐mm‐thick slice. The resulting echo spacing was 552 μs. A constant 15° flip angle SpSp pulse was applied alternately to the [1‐^13^C]pyruvate and lactate resonances, with 1 s between, giving a TR of 2 s for each metabolite. The 11th pair of acquisitions was used as ^13^C reference scans. *T*_2_‐weighted proton images were acquired using an FSE sequence (4 × 4‐cm^2^ FOV, 256 × 256 matrix) at the same slice position.

### 2.4 Image reconstruction

Images acquired from phantoms and *in vivo* were reconstructed in five different ways: using a ^13^C reference scan^25^; using three integrated ^13^C echoes^31^; using a ^1^H reference scan^27^; using a 1D search for the optimal phase correction (zero‐order term and first‐order term in the *x* direction), which minimized signal in the ghost‐containing background; and using a 2D search (including an additional first‐order term in the *y* direction).^30^ Where reference data were acquired, a phase unwrap was first conducted in the *x*–*ky* hybrid space using Ahn's algorithm,^34^ and linear fitting was then performed to obtain the pair of phase correction coefficients. The missing *k*‐space lines were zero‐filled for each partial *k*‐space acquisition prior to Fourier transformation.

**Figure 2:**
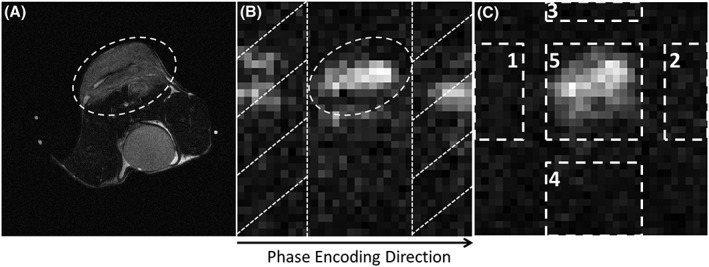
Selection of ghost‐containing image areas, where the signal should be minimized using the proposed workflow. The tumour region was drawn from the proton image A, and the resulting region of interest (ROI) was then applied to the [1‐^13^C]lactate image B. Bands falling outside of the ROI in the phase‐encoding direction (bands with diagonal hatching in B) were considered as ghost‐containing background areas. The image in C, shows the selection of object 5 and background areas 1, 2, 3, 4 in one frame of the ^13^C images, which were used for the measurement of residual ghosting

The proposed workflow is shown in Figure 1. The selection of ghost‐containing regions is illustrated in Figure 2. The tumour region was identified from the proton image (Figure 2A) and used as the desired object in the [1‐^13^C]lactate image (Figure 2B). The selection of these regions for the 1D search method could be simplified as the operator needed only to specify regions beyond the two edges of the tumour in the phase‐encoding direction, minimizing manual intervention. Nyquist ghost correction needed only to be calculated for one image in a series of dynamically acquired images (usually the [1‐^13^C]lactate image with the highest SNR), and the calculated coefficients, α and β, could then be applied to all the *k*‐space lines in all the images in that series. For the phantom studies, α and β were calculated for each individual image, where the proton image was used to identify regions that contained signal. Where there were multiple signal sources, a single region of interest (ROI) was drawn to include all sources of signal. Two bands, at both ends of the phase‐encoding direction (bands with diagonal hatching in Figure 2B), were assumed to be ghost‐containing areas, and the algorithm minimized signal intensity in these areas. An exhaustive search of every combination of α and β was made between values of –π/2 and π/2 (phase error is periodic in π^35^), with 100 increments of each parameter (i.e. 100 × 100 permutations). This had a typical computation time of 15 s on a laptop PC running Matlab 2016a (The Math Works) for images with a matrix size of 32 × 32. For the 2D method, the search was also performed between –π/2 and π/2, again with 100 increments of each of the three parameters and again in the image with the highest signal intensity. The search was conducted for each line in the frequency‐encoding direction, such that signal in the ghost‐containing areas of each line was minimized. With both the ^13^C and ^1^H reference scans, the first‐ and zero‐order correction coefficients were calculated for each line in the phase‐encoding direction. For the ^1^H reference, the coefficients were first corrected for the difference in gyromagnetic ratios before being applied to the ^13^C images. With the integrated ^13^C echoes, a pair of averaged first‐ and zero‐order coefficients was calculated and applied to all the phase‐encoding lines. Phase correction was performed in Matlab using custom‐written scripts.

### 2.5 Calculation of SNR

For SNR calculations in the phantom images, object and noise regions were identified in images phase corrected using a ^13^C reference scan, and then these regions were used for images corrected using the other methods. For images acquired *in vivo*, the object region was obtained in a similar way, from the [1‐^13^C]lactate image with the highest signal, and then applied to all images in the series. The noise standard deviation was calculated from the last [1‐^13^C]pyruvate image.

### 2.6 Calculation of ghosting level

(3) $$\text{Ghosting level}=\left|\frac{\text{mean}\left(\text{region}\;1\right)+\text{mean}\left(\text{region}\;2\right)-\text{mean}\left(\text{region}\;3\right)-\text{mean}\left(\text{region}\;4\right)}{2\times \text{mean}\left(\text{region}\;5\right)}\right|\times 100$$

Mean values were taken from the magnitude signal. Areas 1/2/3/4/5 are indicated in Figure 2C.^36^ Area 5 was drawn on an image corrected using ^13^C reference scan data, to include the whole signal‐containing area (this region could be different for [1‐^13^C]pyruvate and [1‐^13^C]lactate images *in vivo* if, for example, there was significant pyruvate signal from the aorta). This area was then moved by half a FOV in the phase‐encoding direction to define areas 1 and 2, and by half a FOV in the frequency‐encoding direction to define areas 3 and 4. For phantom images with a restricted FOV and multiple signal sources, all five areas were drawn manually.

## 3 RESULTS

Images of the ^13^C phantoms are shown in 3, 4, 5 and a series of [1‐^13^C]pyruvate and [1‐^13^C]lactate images acquired after injection of hyperpolarized [1‐^13^C]pyruvate into a tumour‐bearing mouse are shown in Figure 6.

**Figure 3:**
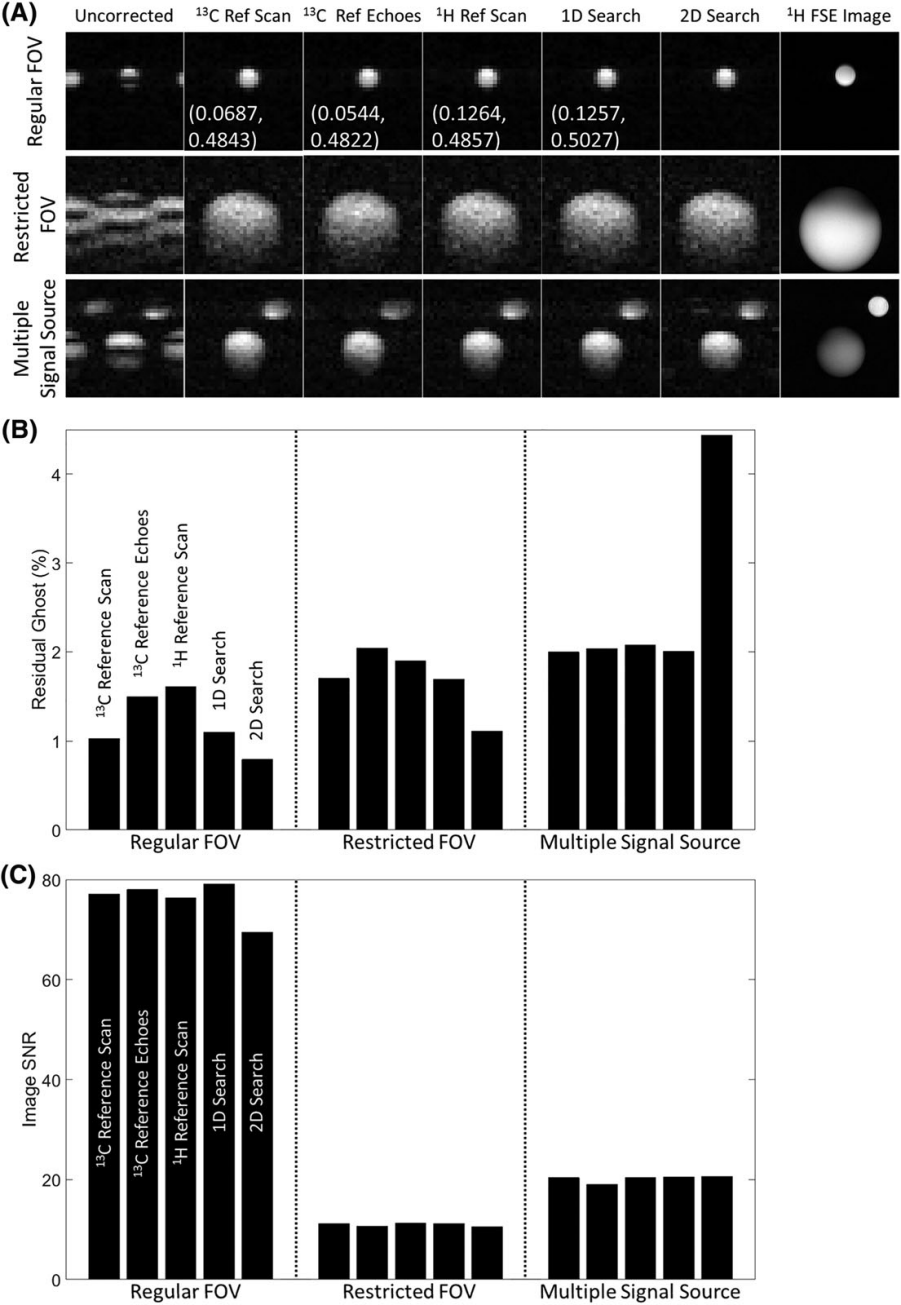
^13^C images of [1‐^13^C]lactate‐containing phantoms at thermal equilibrium, phase corrected using the proposed work flow [one‐dimensional (1D) and two‐dimensional (2D) searches] and the indicated methods (^13^C reference scan, ^13^C reference echoes, ^1^H reference scan). A, Images acquired using a regular field of view (FOV), restricted FOV and from two separate phantoms, and phase corrected using the indicated methods. The measured values for α and β are shown on the images acquired with a regular FOV, except for that corrected using a 2D search, where a different phase error model was used. The difference in the β values between the 1D search and the other methods resulted from the different step size in the search. FSE, fast spin echo. Residual ghosting B, and signal‐to‐noise ratio (SNR) C, were measured for each of the phase correction methods under three different image acquisition conditions. The ghosting levels before correction were 63.2%, 30.8% and 36.6% for the regular FOV, restricted FOV and multiple signal source cases, respectively

**Figure 4:**
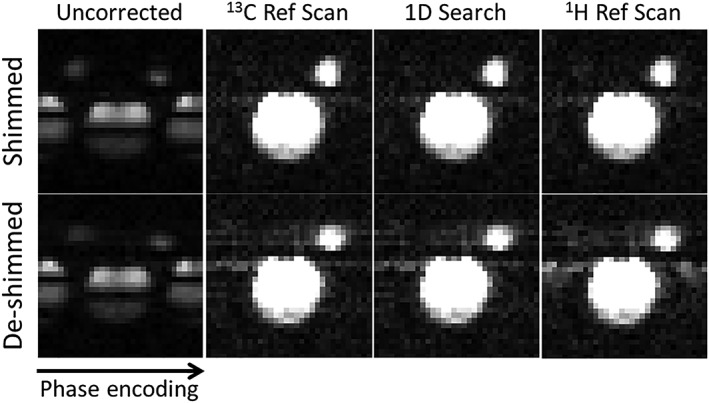
Comparison between the proposed workflow, the ^13^C reference scan method and the ^1^H reference scan method. Images were acquired from a dual phantom with a well‐shimmed magnetic field and in the presence of magnetic field inhomogeneity, where a 6 mT/m^2^ *xy* gradient was applied using the *xy* shim, and the images were phase corrected using the indicated methods. 1D, one‐dimensional

**Figure 5:**
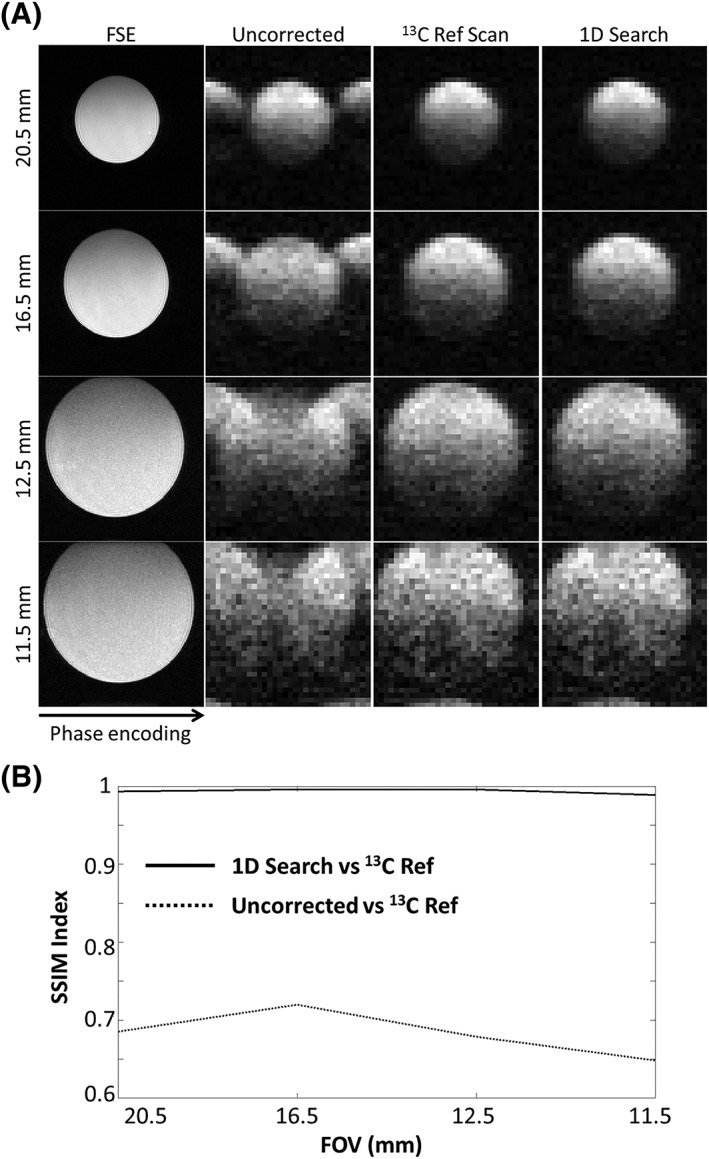
Performance of the proposed workflow with decreasing fields of view (FOVs) (20.5 mm to 11.5 mm) when compared with images corrected using a ^13^C reference scan A. Images were acquired from a centrally placed 10.5‐mm‐diameter phantom. The ^1^H fast spin echo (FSE) images provided a positional reference. The similarities of the images acquired using the workflow and a ^13^C reference scan were measured using the structural similarity (SSIM) index and are shown in B. SSIM = 1 indicates identical images. 1D, one‐dimensional

**Figure 6:**
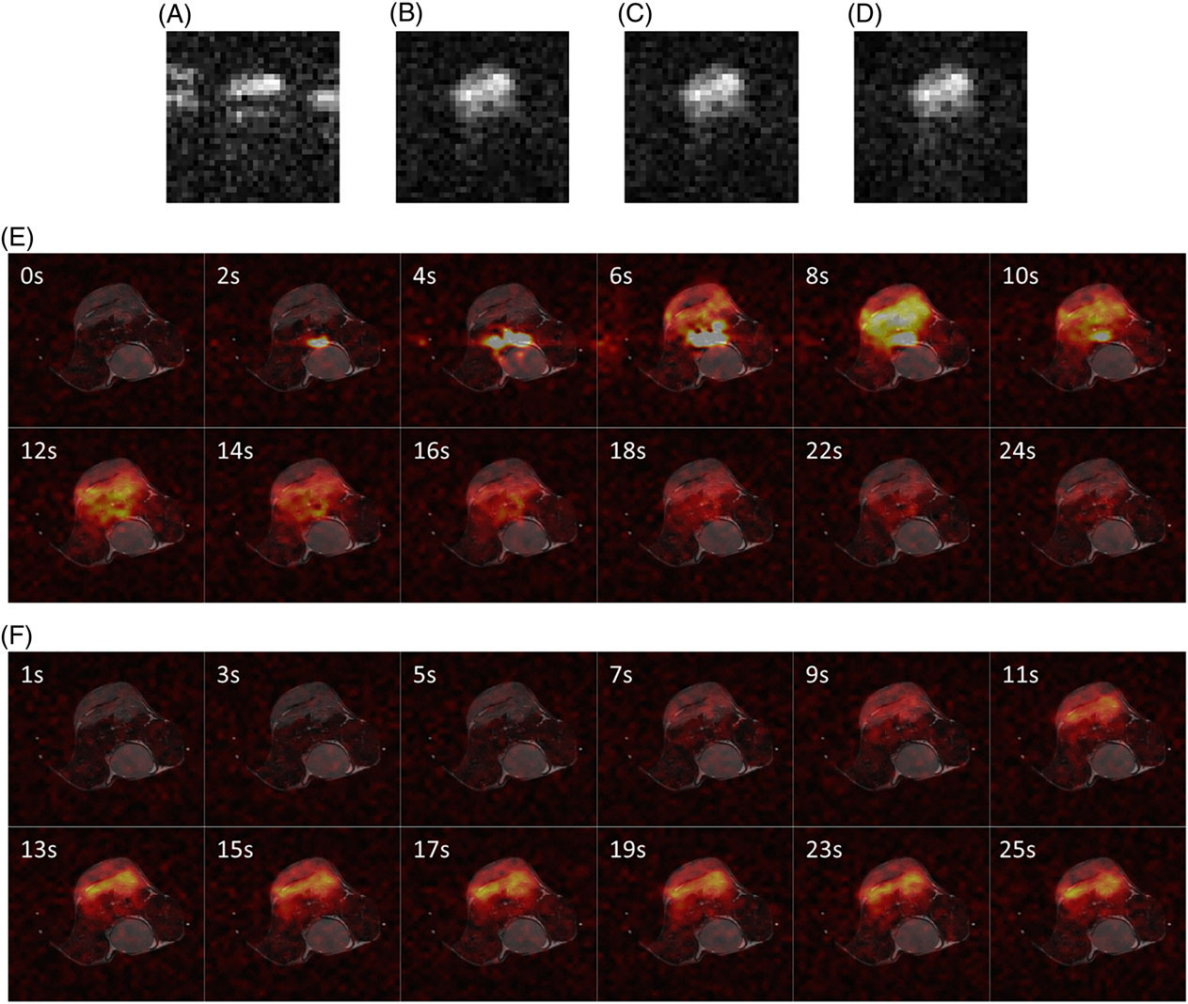
Representative images acquired following the injection of hyperpolarized [1‐^13^C]pyruvate into an EL4 tumour‐bearing mouse. The [1‐^13^C]lactate image acquired at 10 s was used to search for the phase error correction coefficients. Images show a slice reconstructed without phase correction A, phase corrected with ^13^C reference scan data B, corrected with a one‐dimensional (1D) search C, and corrected with a two‐dimensional (2D) search D. E, and F, show dynamically acquired [1‐^13^C]pyruvate E, and [1‐^13^C]lactate F, images, which were corrected using the phase error correction coefficients obtained from C. Only data acquired from the first 25 s are shown. It should be noted that images acquired at 23 and 24 s were used as ^13^C reference scans and are not shown. ^13^C signals in E, and F, were normalized to the pixel with the highest intensity in the [1‐^13^C]pyruvate images

For the phantoms, the 1D search method resulted in images of similar quality to those obtained using a ^13^C reference scan and integrated ^13^C reference echoes, with a large FOV, a restricted FOV and with multiple signal sources (Figure 3A). A quantitative comparison of residual ghosting showed similar ghost removal using all three methods, except that the 2D search almost doubled the residual ghosting, in comparison with the other methods, when there were multiple signal sources (Figure 3B). The images obtained using the proposed workflow with a 1D search showed a similar SNR compared with all the other methods and under all conditions (Figure 3C). Compared with the ^13^C reference scan method, residual ghosting was 1.3% *versus* 1.2% under well‐shimmed conditions and 1.6% *versus* 1.5% under de‐shimmed conditions (Figure 4). However, the images were better than the images corrected using ^1^H reference data when the magnetic field homogeneity was degraded: residual ghosting was 1.4% under well‐shimmed and 3.8% under de‐shimmed conditions (Figure 4) in images corrected using ^1^H reference data.

The influence of FOV on the images corrected using the 1D search and direct ^13^C reference scan methods is shown in Figure 5A. Images corrected using either method were effectively identical. Image similarity was assessed using the structural similarity (SSIM) method^27^, ^37^ (Figure 5B; SSIM = 1 indicates identical images), which describes image similarities based on their luminance, contrasts and structures.

**Figure 7:**
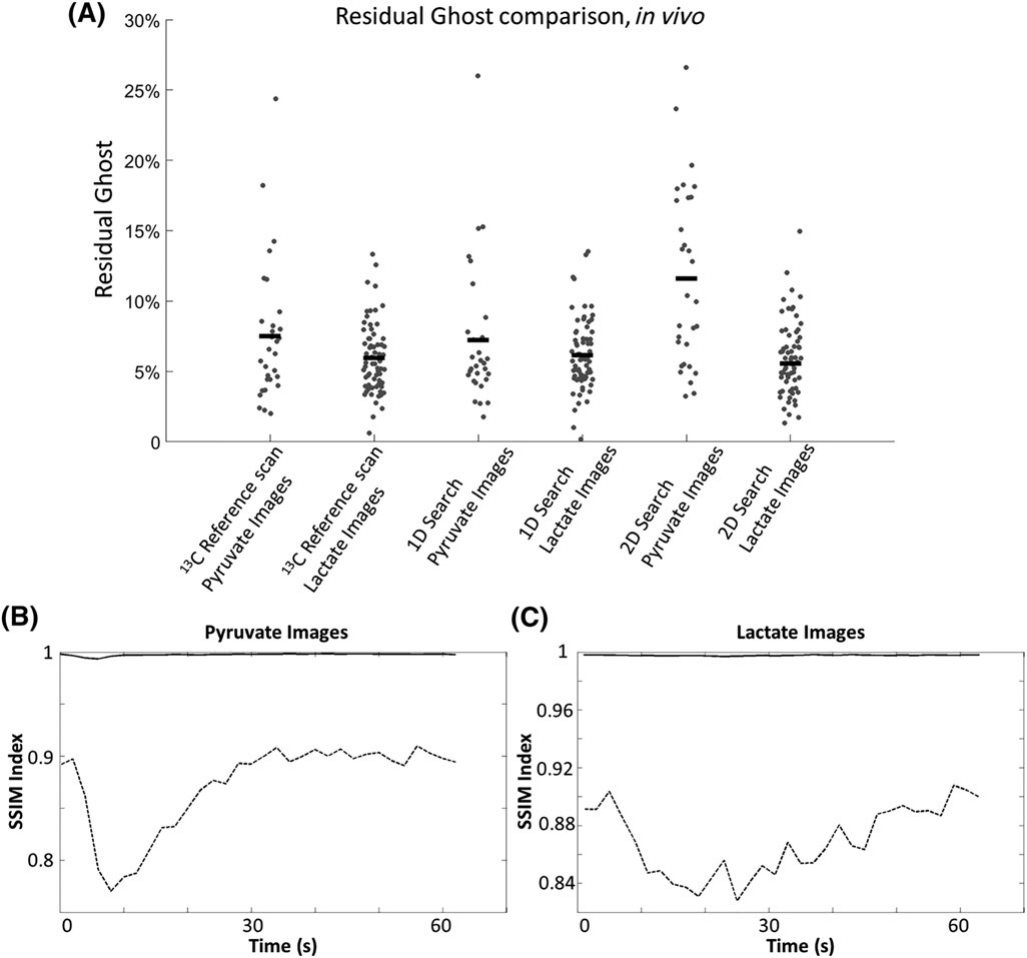
A, Residual ghosting level in the lactate and pyruvate images acquired *in vivo*, where the images were phase corrected using a ^13^C reference scan, one‐dimensional (1D) search and two‐dimensional (2D) search. Both searches were performed on the [1‐^13^C]lactate image with the highest signal‐to‐noise ratio (SNR). The horizontal bars represent the mean value for that data cohort. The similarities (indicated with solid lines) between the images corrected with the ^13^C reference scan and the images corrected using a 1D search were measured for both the [1‐^13^C]pyruvate B, and [1‐^13^C]lactate C, images taken from a representative *in vivo* dataset [structural similarity (SSIM) indices ≈ 1]. The dotted lines show the similarities between the uncorrected images and the images corrected using reference scans

Images acquired *in vivo* were phase corrected using a ^13^C reference scan and 1D/2D searches. For the [1‐^13^C]lactate images, 23 time points, and for the [1‐^13^C]pyruvate images, 10 time points, were selected from the dataset of each of three mice. Images at earlier and later time points were excluded as they were dominated by noise (Figure 6). The 1D and 2D search algorithms gave similar levels of ghosting to the ^13^C reference scan method (Figure 7A). The similarity of dynamic [1‐^13^C]pyruvate and [1‐^13^C]lactate images, from an *in vivo* dataset, and phase corrected using the ^13^C reference scan or 1D search methods, was demonstrated using the SSIM index (Figure 7B, C).

**Figure 8:**
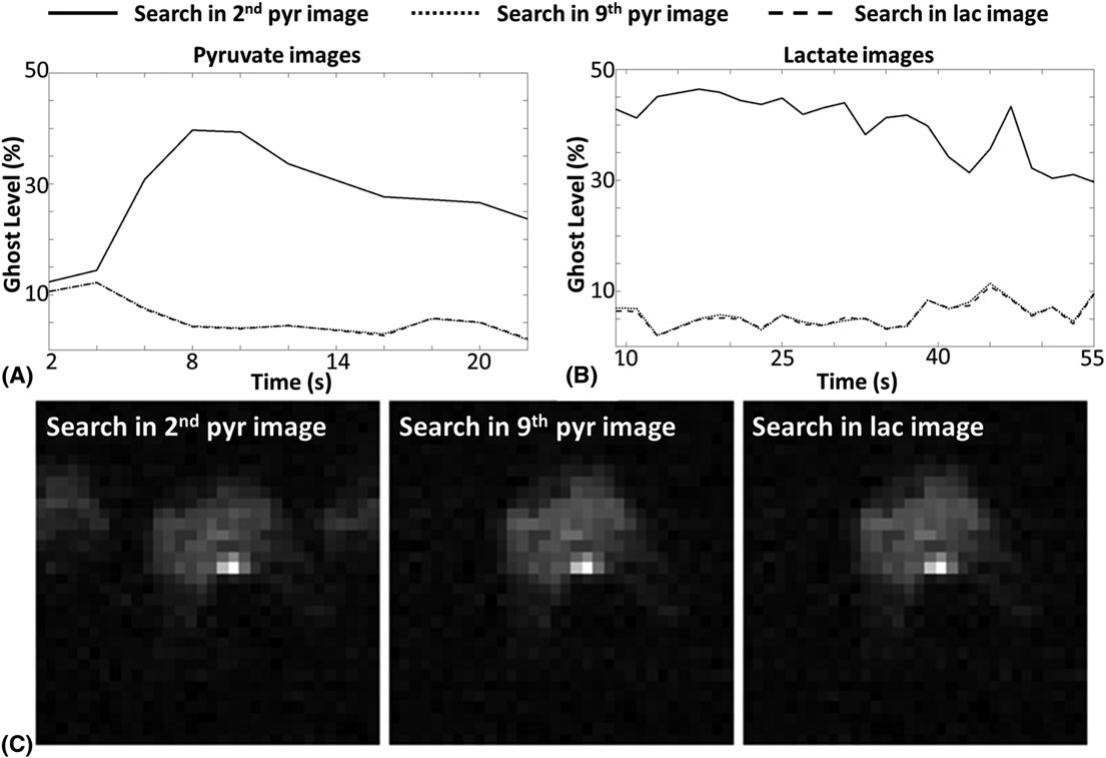
Representative measurements of residual ghosting in [1‐^13^C]pyruvate A, and [1‐^13^C]lactate B, images. The phase corrections were performed based on a one‐dimensional (1D) exhaustive search in the [1‐^13^C]lactate image with the highest signal‐to‐noise ratio (SNR), a [1‐^13^C]pyruvate image acquired at an early stage (2 s) and a [1‐^13^C]pyruvate image acquired at a late stage (16 s) following injection. Representative images from each dataset are displayed in C

Phase correction using an exhaustive search in a late‐stage [1‐^13^C]pyruvate image (ninth image) yielded results similar to those using a search in the [1‐^13^C]lactate image with the highest SNR, whereas a search in an early‐stage [1‐^13^C]pyruvate image (second image) led to considerable residual ghosting (Figure 8). When the sum of the [1‐^13^C]pyruvate images collected over the time course was used, similar phase correction results were obtained with 6.2 ± 2.6% [mean ± standard deviation (SD), 69 images in total, 23 images from each of three mice] and 7.2 ± 5.0% (30 images in total, 10 images from each of three mice) residual ghosting in [1‐^13^C]lactate and [1‐^13^C]pyruvate images, respectively. Similarly, an exhaustive search in the sum of the [1‐^13^C]lactate images gave 6.0 ± 2.5% (*n* = 69) and 8.1 ± 5.0% (*n* = 30) residual ghosting in the [1‐^13^C]lactate and [1‐^13^C]pyruvate images.

**Figure 9:**
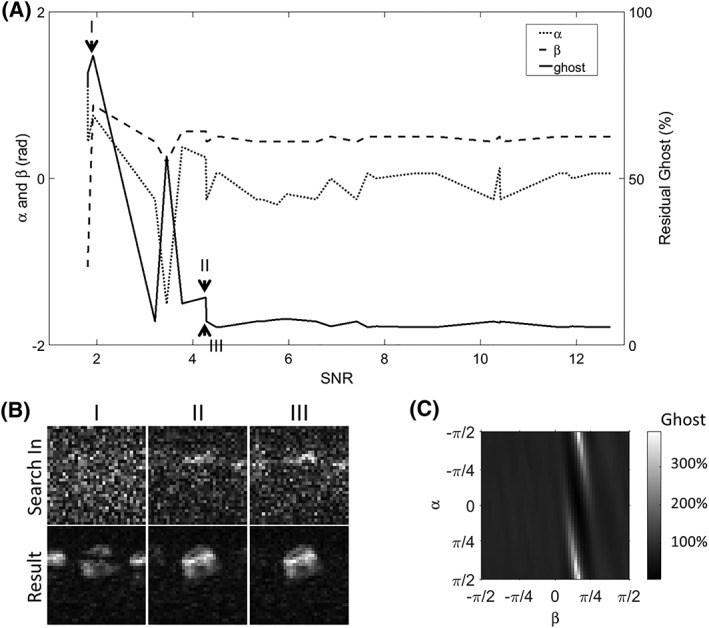
A, the tolerance of the one‐dimensional (1D) search to differences in signal‐to‐noise ratio (SNR) was examined using the lactate images from one representative *in vivo* dataset. The search was performed on each of the lactate images (with SNRs ranging from 1.8 to 12.7) and the pair of phase correction coefficients obtained was then applied to the lactate image with the highest SNR. The resulting image was free of ghosting until the SNR of the individual lactate images was less than 4.7. The α and β pairs obtained from the searches in each image are also plotted. B, Representative lactate images with different SNRs (top row), which were used to determine α and β pairs, and the corresponding phase‐corrected lactate image with the highest SNR. C, Residual ghosting in the same image when corrected using different pairs of phase correction coefficients

To evaluate the tolerance of the proposed workflow to differences in SNR, a 1D search was performed on each of the lactate images from an *in vivo* dataset. The coefficients obtained were then applied to the lactate image with the highest SNR. Residual ghosting in this latter image and the coefficients obtained from the individual images were then plotted against the SNR of the individual images (Figure 9A, B). The workflow yielded a ghost‐free image when the SNR of the image from which the coefficients were calculated was greater than 4.7. Below this value, the coefficients obtained appeared to be random. We also applied a range of coefficient pairs (from –π/2 to π/2 for each coefficient) to the same [1‐^13^C]lactate image with the highest SNR, and found that the algorithm was relatively tolerant to a range of values for α provided that β was measured correctly, as was also observed using the other phase correction methods (Figure 3).

The 1D search method would have had a clear SNR benefit *in vivo* when compared with phase correction using integrated ^13^C echoes. Although we did not collect integrated reference ^13^C echoes *in vivo*, with the reference scan data we can calculate the signal loss that would have resulted from their collection by comparing the signal intensities in the echo acquired at the centre of *k* space and three echoes later. The 1D search method showed an SNR benefit of 17.0 ± 9.1% (*n* = 3) for [1‐^13^C]pyruvate and 15.3 ± 8.7% (*n* = 3) for [1‐^13^C]lactate.

## 4 DISCUSSION

The proposed method reliably removed Nyquist ghosting in ^13^C echo planar images acquired from phantoms and *in vivo*, showing similar removal of ghosting as the direct ^13^C reference scan method (Figures 3, 4, 5, 6, 7). In our experience, the remaining blurring and ringing artefacts in the phase‐encoding direction in Figure 3A are the result of zero‐filling during image reconstruction. Compared with conjugate reconstruction for partial *k*‐space acquisition, zero‐filling improves the SNR, but causes image blurring and Gibbs ringing. Residual artefacts in the phase‐encoding direction in [1‐^13^C]pyruvate images acquired *in vivo* at 4 and 6 s (Figure 6E) were primarily from high‐intensity signals in blood vessels, which were undergoing rapid flow and could not be accurately phase corrected. The same artefacts were observed in images corrected using a ^13^C reference scan. The proposed workflow does not require the sacrifice of a time point in the limited image acquisition time window, potentially giving a more accurate estimate of kinetic rate constants. Although not important in this case, where signals from pyruvate and lactate were relatively long lived, this will be more important when there is more rapid polarization decay.

### 4.1 1D search and 2D search

A 2D search offered no advantage over a 1D search. Residual ghosting levels in *in vivo* [1‐^13^C]pyruvate images were 6.3 ± 3.4% (*n* = 30) and 11.4 ± 5.9% (*n* = 30) for the 1D and 2D searches, respectively, and, for the *in vivo* [1‐^13^C]lactate images, were 6.0 ± 2.5% (*n* = 69) and 5.6 ± 3.0% (*n* = 69), respectively. However, noise levels were higher in images corrected using the 2D search because of matrix inversion in the phase correction algorithm. For a modern magnetic resonance scanner, where the gradient coils are well shielded and cross‐term eddy currents are minimized, correction in both the readout and phase‐encoding directions is not required.^29^, ^30^ In the phantom experiments, where there were multiple signal sources, the 2D search method led to more residual ghosting than the 1D search method, especially from the small phantom. This was because the 2D search relied on local ghost minimization for each line in the frequency‐encoding direction, which could be biased in lines where little ghosting was present. This problem could be resolved by explicitly drawing ROIs for each signal source. When this was performed, the residual ghosting was reduced to 1.9%, which is similar to that obtained with all the other methods. With oblique plane imaging, the 2D search method may be needed to correct for *k*‐space displacement in both the *kx* and *ky* directions. However, even in this situation, the 1D search method should perform better than the abovementioned reference scan methods as it directly minimizes ghosting in the image domain.

### 4.2 1D search and other phase correction methods

The 1D search is based on a linear phase error model that is similar to that of the widely used reference scan method, which has been proven to be robust on modern clinical systems. The 1D search renders a reference proton scan^27^ unnecessary, which can be compromised by differences in the spatial location of the ^1^H and ^13^C signals. Eddy currents, a major source of Nyquist ghosting, are spatially dependent, and a mismatch in location may render the proton reference scan less than optimal for phase correction (see Figure 4). This mismatch may arise because the ^1^H image has only one‐quarter of the FOV of the ^13^C image in the readout direction. Although a four times larger receiver bandwidth could be used in the ^1^H image, in order to keep the same readout FOV, the high sampling rate needed may exceed hardware capability (a large receiver bandwidth is already used for EPI to reduce image distortion). For example, a 250‐kHz bandwidth in ^13^C imaging will require a 1‐MHz receiver bandwidth in the ^1^H reference scan. Moreover, if the same slice selection gradients are used in acquisition of the ^1^H reference, the slice thickness will also be one‐quarter of that in the ^13^C image. A smaller (one‐quarter) slice selection gradient could be used, but then the eddy currents would be different. As these eddy currents may interfere with the eddy currents generated by the readout gradients, different phase errors may be measured in the ^1^H reference than are actually present in the ^13^C image.

An EPI sequence with integrated ^13^C reference echoes^28^, ^31^ does not use up a dynamic time point, acquires reference data directly from the ^13^C signals and requires no operator intervention. However, it sacrifices image SNR by prolonging TE. The method proposed here has therefore a substantial SNR benefit *in vivo* over the integrated reference echoes method. Although clinical systems usually have lower field strengths than the 7 T used here, and therefore longer *T*_2_*, this SNR benefit may still hold in clinical applications because of the slower gradient slew rates and the potential need for a larger acquisition matrix to cover a larger FOV. The integrated reference echoes may be inserted at the end of the acquisition train so that the SNR of the image would not be sacrificed; however, this may result in very low signal from the reference echoes.

Reference data could be acquired from a ^13^C phantom. However, a phantom further complicates the spectrum and places more restrictions on SpSp pulse design. Using an image from the phantom for phase correction will also potentially suffer from the same problems as using a proton reference image, where differences in spatial location and eddy currents could lead to suboptimal calculation of the phase correction coefficients.

The exhaustive search method has not been widely used in the clinic for proton MRI, mainly because many imaging objects cover most of the FOV and, if there is a large overlap between object and ghosting, the exhaustive search method can sometimes lead to heavily biased results. This is not the case for ^13^C imaging because the ^13^C signals are usually localized. This was the case here, where labelled lactate was observed predominantly in the tumour, but is also the case for other hyperpolarized ^13^C‐labelled imaging agents, such as the perfusion markers ^13^C‐labelled urea^38^, ^39^ and [1‐^13^C]2‐methylpropan‐2‐ol (t‐butanol).^40^, ^41^ For perfusion imaging, searches in early‐stage images may lead to biased correction coefficients because of rapid motion of the marker in the blood vessels, as is indicated by the results shown in Figure 8. These effects could potentially be reduced using EPI‐based sequences designed to reduce flow artefacts.^42^ Motion artefacts could also be minimized by summing images over the time course. As shown here, a search based on the summed [1‐^13^C]pyruvate images yielded similar phase correction coefficients to those determined from the [1‐^13^C]lactate image with the highest SNR.

### 4.3 Robustness

The proposed method works well with a restricted FOV, provided that the target metabolite signal does not cover the whole FOV, and also with multiple signal sources. The method worked well even when the object filled up to ≈90% of the FOV in both the phase‐ and frequency‐encoding directions. Images of a centrally placed 10.5‐mm‐diameter phantom phase corrected using a 1D search were similar to those phase corrected using a ^13^C reference scan between FOVs of 11.5 and 20 mm (SSIM > 0.98) (Figure 5). In unusual cases in which no major localized signal is present and multiple signal‐containing regions are located close to both ends in the phase‐encoding direction, such that there is no ghosting outside the selected object region, an entropy‐based convergence criterion^29^, ^35^ may be needed. However, an entropy‐based criterion was not used in our implementation because it can result in an image shifted by half a FOV when large eddy currents are present, as the entropy is the same for the original and shifted images.

The second step in Figure 1A is just one of the possible implementations of the proposed workflow. The workflow is relatively tolerant to the selection of images in which to perform the 1D exhaustive search. Similar correction results were obtained from a search in the [1‐^13^C]lactate image with the highest signal intensity, from a late‐phase [1‐^13^C]pyruvate image and from [1‐^13^C]pyruvate and [1‐^13^C]lactate images that were summed over the entire time course. The workflow is also tolerant to a wide range of SNR conditions (Figure 9A).

Gross shifts and distortions in the ^13^C image caused by *B*_0_ field drift and inhomogeneity may lead to a spatial mismatch between ^13^C and ^1^H images (i.e. the object in the ^13^C image falls partially outside the ROI drawn in the ^1^H image). Fortunately, the relatively small ^13^C gyromagnetic ratio and small acquisition matrix (hence high bandwidth in the phase‐encoding direction) make it less prone to off‐resonance effects. Ramp sampling can also help by further increasing the phase‐encoding bandwidth. For example, the 552‐μs echo spacing used here results in an immunity to a *B*_0_ shift of ±57 Hz, which can be increased to approximately ±100 Hz when ramp sampling is used with the same gradient amplitude and slew rate. In comparison, the ^13^C linewidth of [1‐^13^C]lactate *in vivo* is usually around 40 Hz on our system. Dynamic central frequency measurements could be incorporated into the experiment by using a simple pulse‐acquire sequence with a small flip angle, although, in our experience, this is unnecessary as the central frequency does not vary much (usually a few Hertz) after the injection of the hyperpolarized medium.

### 4.4 Compatibility

The proposed workflow could readily be applied to 3D EPI, where the 1D search could be performed per slice and the correction coefficients obtained applied to each *kx*–*ky*–*z* hybrid *k*‐space (the hybrid *k*‐space is obtained by Fourier transformation of the 3D *k*‐space in the *kz* direction). The method is also compatible with multi‐channel receive coils. Provided that accelerated acquisition is not used, the search need only be on data from a single channel. When there are strong eddy currents in *y* or *z*, a search may be needed in the image from each channel. In the case of parallel imaging, the phase correction coefficients in each iteration of the search should be implemented in the sensitivity map in a similar way to that described by Chen et al.^30^ The image should then be reconstructed with this modified sensitivity map for each iteration of the search.

## 5 CONCLUSIONS

We have developed a referenceless EPI workflow for hyperpolarized ^13^C‐labelled metabolites. This workflow is as capable of removing Nyquist ghosting as a ^13^C reference scan, at no cost of extra data acquisition during dynamic imaging, and would be straightforward to implement in clinical imaging.
